# Maze Test Score Adjustments When Using Non-Dominant Hand in Fitness-To-Drive Assessments

**DOI:** 10.1093/geroni/igaa057.1686

**Published:** 2020-12-16

**Authors:** Carolyn Unsworth, Gemma Hext, Anne Baker, Matthew Browne

**Affiliations:** 1 Central Queensland University, Melbourne, Victoria, Australia; 2 Australian Catholic University, melbourne, Victoria, Australia; 3 Australian Catholic University, Melbourne, Victoria, Australia; 4 CQUniversity, Bundaberg, Queensland, Australia

## Abstract

Older drivers with health impairments may be required to undertake fitness-to-drive assessments. Scores on the Occupational Therapy-Drive Home Maze Test (OT-DHMT) can contribute to fitness-to-drive recommendations. The OT-DHMT is a short, timed maze test that has been shown to be valid and reliable, and norms are available for completion with a driver’s dominant hand. However, the validity of a person’s score when using their non-dominant hand to complete the test, for example following stroke, is unknown. This study aimed to determine if a person’s OT-DHMT score time (in seconds) requires adjustment when completed with a non-dominant hand. The OT-DHMT was administered with a normative sample of 150 participants, aged 21-81 years (mean=48.6,SD=19.38). Overall, OT-DHMT score times were significantly faster when using a dominant (M=15.73) compared with non-dominant (M=17.64) hand, d=1.91 (CI 1.13, 2.69), t= 4.84,p< .01. Employing a generalised weighted least squares regression model indicated that multiplying a driver’s non-dominant hand time by .833 seconds for drivers aged ≤60, and by .929 seconds for drivers aged 61+ can approximate dominant hand completion times. Adjusted scores can then be compared against normed scores to aid fitness-to-drive recommendations. The adjustment required for people aged ≤60 is larger than for older people, reinforcing previous findings that younger people have faster OT-DHMT completion times. These findings support the clinical utility and vaildity of using the OT-DHMT with older people undergoing fitness-to-drive assessment who may be required to use their non-dominant hand due to conditions such as stroke, arthritis or amputation.

